# LPN: Label-Enhanced Prototypical Network for Legal Judgment Prediction

**DOI:** 10.3390/e25101398

**Published:** 2023-09-29

**Authors:** Junyi Chen, Yingjie Han, Xiabing Zhou, Hongying Zan, Qinglei Zhou

**Affiliations:** 1 School of Computer and Artificial Intelligence, Zhengzhou University, Zhengzhou 450001, China; jychen@gs.zzu.edu.cn (J.C.);; 2 School of Computer Science and Technology, Soochow University, Suzhou 215006, China

**Keywords:** legal judgment prediction, document classification, prototypical network, label-enhanced

## Abstract

As one of the most critical tasks in legal artificial intelligence, legal judgment prediction (LJP) has garnered growing attention, especially in the civil law system. However, current methods often overlook the challenge of imbalanced label distributions, treating each label with equal importance, which can lead the model to be biased toward labels with high frequency. In this paper, we propose a label-enhanced prototypical network (LPN) suitable for LJP, that adopts a strategy of uniform encoding and separate decoding. Specifically, LPN adopts a multi-scale convolutional neural network to uniformly encode case factual description to capture long-distance features of the document. At the decoding end, a prototypical network incorporating label semantic features is used to guide the learning of prototype representations of high-frequency and low-frequency labels, respectively. At the same time, we also propose a prototype-prototype loss to optimize the prototypical representation. We conduct extensive experiments on two real datasets and show that our proposed method effectively improves the performance of LJP, with an average F1 of 1.23% and 1.13% higher than the state-of-the-art model on two subtasks, respectively.

## 1. Introduction

Legal judgment prediction (LJP) refers to predicting the judgment result based on the factual description of cases and established statutes [1]. Depending on the results, it is usually divided into three subtasks, i.e., relevant article prediction, charge prediction, and penalty term prediction. In real-world scenarios, this task is exclusively undertaken by legal practitioners who have undergone years of professional training. Each step of making a judgment is time-consuming process that requires a solid foundation in the legal field. This places a significant burden on the restricted pool of legal practitioners. In Brazil, an astounding 1.66 million cases related solely to financial matters are filed each day, with the majority of cases taking years to resolve [2]. Whereas if there is an automated LJP system as an auxiliary tool, it will significantly boost the productivity of legal practitioners and alleviate the backlog of cases. In addition, since it is constructed based on previous cases, it can also effectively improve the fairness and consistency of judgments [3].

**Figure 1:**
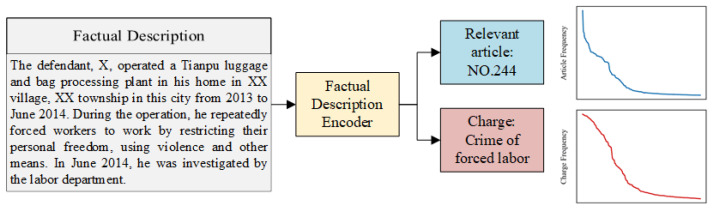
An example of LJP, the line graph on the right shows the frequency of each article and charge in the CAIL-Small dataset.

With the vigorous development of deep learning techniques, there is growing interest in LJP. Existing approaches usually regard LJP as a document classification task and have proposed some commendable methods. For example, the topological multi-task framework proposed by Zhong et al. [4] effectively utilized the principle of information consistency among sub-tasks and has been widely adopted by subsequent researchers. However, there is a notable challenge in LJP, i.e., a large number of case categories and severely imbalanced distribution make it difficult for the model to learn the features of cases with limited samples (few-shot cases). As illustrated in Figure 1, there is an obvious long-tail distribution phenomenon about legal articles and charges in cases, and the few-shot cases account for more than 50%. In reality, however, legal practitioners tend to spend more time on these uncommon cases. It is urgent to develop LJP models to assist them in improving this situation.

To address this issue, Hu et al. [5] introduced manually labeled attributes to help predict few-shot charges, while it has poor transferability. Recently, Liu et al. [6] divided cases into high-frequency and low-frequency according to the frequency of legal articles or charges and then fed them into the model simultaneously as tuples. Inspired by them, we divide legal cases into the “head set” (high-frequency) and “tail set” (low-frequency). Subsequently, we employ a strategy of unified encoding and separate decoding for learning. Unlike them, we explore improving few-shot performance with a simple and effective prototypical network [7]. Specifically, we first adopt a multi-scale convolutional neural network (CNN) to encode the factual description, so that realize the transfer of the underlying features learned in large samples to few-shot ones. Subsequently, the features of these two sets are then decoded independently using two decoders. These decoders fuse the semantic information from established statutes (as shown in Table A1) with a prototype network to effectively learn the corresponding prototype representation for each category. Finally, we achieve LJP by similarity measures between cases and each prototype.

To evaluate the efficacy of the proposed model, we conduct experiments on two real-world datasets constructed from Chinese criminal cases. As a consequence, we make the following four primary contributions:

1. We divide the dataset into the “head set” and “tail set”, then decode them separately to alleviate the problem of model learning bias caused by data imbalance.
2. We propose a label-enhanced prototypical network(LPN) for LJP, which incorporates established article description information as label enhancement to learn the prototype representation of labels.
3. We design a prototype-prototype loss to optimize the learning process according to the structure of LPN.
4. Experimental results on two datasets show that our proposed method effectively improves the performance of LJP, and the average F1 on the two subtasks is 1.23% and 1.13% higher than the state-of-the-art model, respectively. Especially in the few-shot data where LPN leads by a wider margin.

## 2. Related Work

This section reviews the existing literature related to our research and points out the differences between previous studies and our work.

### 2.1. Legal Judgment Prediction

LJP has a long research history. Early works employed mathematical statistics and rule-based methods, e.g., Kort et al. [8] and Nagel et al. [9]. These methods make the prediction results interpretable but require manual rule-making and are poorly transferable.

With the successful usage of neural network methods on natural language processing [10,11], researchers have mostly adopted neural models to solve LJP. For example, Bao et al. [12] proposed an attentional neural network, which predicted the legal article first and then employed the article to help improve charge prediction. Chen et al. [13] proposed a multiple residual article-wise attention network, which adopted a multi-scale convolutional network to encode factual descriptions, and incorporated label information instead of just using labels as the index to achieve LJP. Considering the dependencies among multiple subtasks, Zhong et al. [4] formalized the dependencies as a Directed Acyclic Graph(DAG) and proposed a topological multi-task learning framework to realize the three subtasks predictions, which is very representative and effective. Subsequently, Yue et al. [14] proposed a circumstance-aware framework that utilized the results of intermediate subtasks to separate the factual description into different circumstances and exploited them to make the predictions of other subtasks. Furthermore, Chen et al. [15] improves the performance of LJP by exploiting the consistency constraint relations of the three subtasks.

However, none of them considered the problem of severely imbalanced label distribution in the data of LJP. Hu et al. [5] achieved few-shot charge prediction by introducing some attributes constructed manually, but it has poor transferability. And Han et al. [16] proposed BERT-Attention based on easy data augmentation techniques to achieve few-shot charge prediction, which added a large amount of data to alleviate the imbalanced distribution of the original data, but it is easy to cause overfitting. Recently, Liu et al. [6] constructed case triples as input which contain two similar cases and one dissimilar case, using the relationship and frequency information of cases to optimize model learning, and achieved good results. Taking inspiration from these approaches, we divide the dataset into the “head set” and the “tail set” based on the label frequency, constructing separate decoders for each. Thereby alleviating the problems caused by data imbalance.

### 2.2. Few-Shot Classification

Few-shot classification is a classification task that aims to train models to perform well for classes with very few training samples. Some efficient algorithms have been proposed for this task, such as gradient-based methods [17] and metric-based methods [7,18]. For the metric-based methods, their basic idea is first to learn a feature mapping function that projects samples into an embedding space, then compute their relations through some metrics for classification. Among them, the prototypical network is one of the most popular methods for few-shot learning due to its simplicity and effectiveness.

Based on the above two methods, transferring the existing knowledge to realize the few-shot classification has been proven effective [19]. Recently, Mueller et al. [20] and Liu et al. [21] improved few-shot classification performance by exploiting the semantic information inherent in labels. Specifically, Mueller et al. [20] proposed a method for incorporating label semantics into generative models during pretraining to improve few-shot intent recognition. Liu et al. [21] proposed a novel label-enhanced prototypical network for multi-label few-shot aspect category detection, but only for n-way k-shot datasets. Different from them, we not only transfer the underlying knowledge learned from large samples to few-shot ones but also improve the prototype network fusing label semantic information to get rid of the n-way k-shot limitation.

## 3. Method

**Figure 2:**
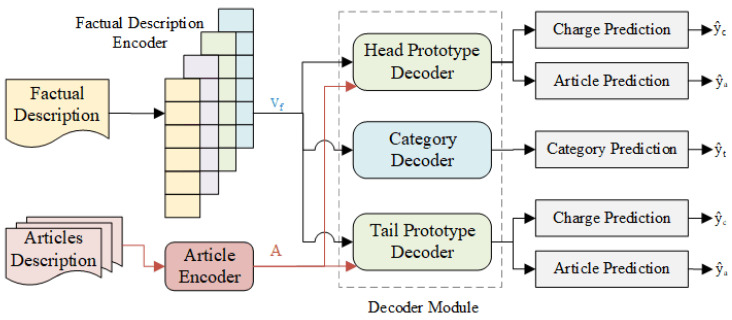
Overview of our proposed model (LPN).

This section introduces the framework of our proposed model LPN (as shown in Figure 2). Since we improve LJP performance from the perspective of optimizing the representation of few-shot legal cases, we only solve the two sub-tasks of relevant article prediction and charge prediction. LPN consists of the following components, i.e., the factual description encoder composed of multi-scale CNN, articles encoder for label enhancement, category decoder as a switch that aims to distinguish the head and tail cases, head and tail decoder based on prototype network, etc. Next, we will present them in detail. Note that we denote the matrix in bold.

### 3.1. Task Formalization

Like most existing studies [6,22], we regard LJP as a document classification task. The main mathematical notations in the paper are shown in Table 1. The training legal cases set can be denoted as $\Gamma ={\left(S_{i}^{d},Y_{i}\right)}_{i=1}^{N}$, where *N* is the number of legal cases. As mentioned above, we aim to predict the relevant article and the charge for each case simultaneously, so $Y_{i}=\left\{y_{i}^{a},y_{i}^{c}\right\}$, where $y_{i}^{a}\in Y^{a}$ and $y_{i}^{c}\in Y^{c}$. Our goal is to learn a classifier *f* from Γ that can predict the judgment results on data with unknown labels, i.e., $f\left(S_{u}^{d},Y^{a},Y^{c}\right)\Rightarrow \left\{\hat{y_{u}^{a}},\hat{y_{u}^{c}}\right\}$, where $S_{u}^{d}\notin \Gamma$.

### 3.2. Factual Description Encoder

First, we convert each word of factual description $S_{i}^{d}$ into its word embedding, so that gets its embedding representation $X_{i}\in R^{ld\times k}$, where *k* is the dimension of word embedding and ld is the length of $S_{i}^{d}$. Since the core information of factual description is scattered, we employ multiple convolutional operations with different filter sizes to capture rich textual representation. Taking the filter size *s* as an example, the specific calculation is as follows:

(1) $$\begin{array}{cc} c_{s} & =\cap_{j=1}^{ld}W_{s}\cdot X_{i}^{j:j+s-1}+b_{s} \end{array}$$

(2) $$\begin{array}{cc} d_{s} & =max\{c_{1},\ldots ,c_{ld-s+1}\} \end{array}$$

where $\cap_{j=1}^{ld}$ represents a convolution operation from left to right, $W_{s}\in R^{f\times (s\times k)}$ is the trainable parameter matrix, $X_{i}^{j:j+s-1}\in R^{s\times k}$ is a part of $X_{i}$, $b_{s}\in R^{f}$ is the bias vector, $d_{s}\in R^{f}$ is the output of filter *s*, where *f* is the output channel size.

Subsequently, since each filter has the same out-channel size *f*, we concatenate the output obtained with different filter sizes to get the representation of the factual description $v_{f}\in R^{h}$, where *h* is the hidden dimension equals to the number of filters multiplied by *f*.

### 3.3. Established Articles Encoder

The established statutes all have specific descriptions, as shown in Table A1. We encode these descriptions as the label-enhanced information of the legal article, integrating them into the model. Specifically, we use the same method as in Section 3.2 to obtain the matrix representation $A=\left\{A_{1},\ldots ,A_{m}\right\}\in R^{m\times h}$ of all the articles’ description, where *m* is the number of articles. Then we put $A_{i}$ as the initial prototype of article label *i*.

### 3.4. Category Decoder

The category decoder has been shown to be effective in [6], which is designed to distinguish whether a case belongs to the high frequency set or few-shot ones. Its role is not only to transfer the underlying features learned in the “head set” to the “tail set” but also to alleviate the problem of imbalanced distribution of original data.

Specifically, we first count the occurrence frequency of different labels and arrange them in descending order. Next, we establish a threshold variable, denoted as μ, and divide the head and tail sets according to the ratio of μ:1. In our implementation, we use 1 and 0 to distinguish head and tail sets.

(3) $$\begin{array}{cc} v_{t} & =W_{t}^{2}\cdot ReLU\left(W_{t}^{1}\cdot v_{f}\right)+b_{t} \end{array}$$

(4) $$\begin{array}{cc} {\hat{y}}_{t} & =argmax\frac{exp(v_{t})}{\sum exp(v_{t})} \end{array}$$

where $W_{t}^{1}\in R^{h\times h}$, $W_{t}^{2}\in R^{h\times 2}$ and $b_{t}\in R^{2}$ are trainable weight matrix and bias. ${\hat{y}}_{t}$ is the predicted category label.

### 3.5. Head/Tail Prototype Decoder

According to the category label $y_{t}$, we select the corresponding branch to decode the factual description $v_{f}$. Note that the decoders on both head and tail branches have the same structure, but do not share parameters.

#### 3.5.1. Multi-Task Decoder

First, a multi-task framework is required to achieve both article prediction and charge prediction at the same time. We adopt the topological framework proposed by Zhong et al. [4] to obtain the corresponding hidden representations $h_{a}$ and $h_{c}$ of the different subtasks, the detailed calculation process is as follows:

(5) $$\begin{array}{cc} \left[\begin{array}{c} h_{a}\\ c_{a} \end{array}\right] & =LSTMCell\left(v_{f},\left[\begin{array}{c} {\bar{h}}_{a}\\ {\bar{c}}_{a} \end{array}\right]\right) \end{array}$$

(6) $$\begin{array}{cc} \left[\begin{array}{c} {\bar{h}}_{c}\\ {\bar{c}}_{c} \end{array}\right] & =W_{c}*\left[\begin{array}{c} h_{a}\\ c_{a} \end{array}\right]+b_{c} \end{array}$$

(7) $$\begin{array}{cc} \left[\begin{array}{c} h_{c}\\ c_{c} \end{array}\right] & =LSTMCell\left(v_{f},\left[\begin{array}{c} {\bar{h}}_{c}\\ {\bar{c}}_{c} \end{array}\right]\right) \end{array}$$

where $W_{c}\in R^{h\times h}$ and $b_{c}\in R^{h}$ are the trainable weight and bias, ${\bar{h}}_{a},{\bar{c}}_{a}$ are the initial hidden state and the memory cell of the article prediction task, respectively. Finally, we get the decoded representations $h_{a}$ and $h_{c}$ of these two tasks.

#### 3.5.2. Prototype Learning Module

Next, we fuse A get from Section 3.3 to construct two Euclidean spaces for article labels and charge labels, respectively. We adopt Euclidean distance to measure the similarity of the hidden features ha,hc to each label prototype $P_{a_{i}},P_{c_{i}}$ as calculated in Equations (8) and (9). Correspondingly $P_{a_{i}}=A_{i}$ is the prototype representation of article $a_{i}$. $P_{c_{i}}$ is the prototype representation of charge $c_{i}$, which can be learned from LPN. Our goal is to learn prototypical representations for each label, and then predict the relevant article and charge based on these prototypes. The corresponding prediction process is as follows.

(8) $$\begin{array}{cc} d(P_{a_{i}},h_{a}) & =-∥\vec{P_{a_{i}}},\vec{h_{a}}{∥}_{2} \end{array}$$

(9) $$\begin{array}{cc} d(P_{c_{i}},h_{a}) & =-∥\vec{P_{c_{i}}},\vec{h_{c}}{∥}_{2} \end{array}$$

(10) $$\begin{array}{cc} {\hat{y}}_{a}=argmax & \;\frac{exp\;d(P_{a_{i}},h_{a})}{\sum_{i=1}^{m}exp\;d\left(P_{a_{i}},h_{a}\right)} \end{array}$$

(11) $$\begin{array}{cc} {\hat{y}}_{c}=argmax & \;\frac{exp\;d(P_{c_{i}},h_{c})}{\sum_{i=1}^{n}exp\;d\left(P_{c_{i}},h_{c}\right)} \end{array}$$

Finally, we get the predicted article label ${\hat{y}}_{a}$ and charge label ${\hat{y}}_{c}$.

**Figure 3:**
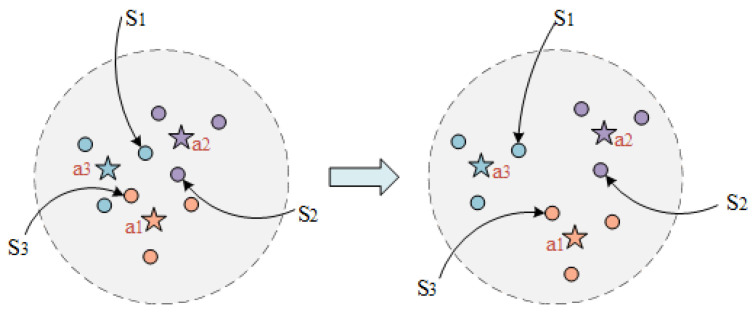
A two-dimensional representation of the prototype of some legal articles and factual description, where five-pointed stars represent the prototype of legal articles, and circles represent factual descriptions.

**Prototype-prototype loss.** Considering the multitude of prototypes to be learned, to ensure their separability in a relatively low-dimensional space, we propose a prototype-prototype loss. This loss function serves to increase the distance between prototypes of different labels, so that the factual descriptions of different labels have enough space to distinguish and reducing the likelihood of confusion. As shown in Figure 3, it is a two-dimensional illustration of the legal article prototypes and factual description, where cases with the same legal article surround their corresponding prototype. Cases $S_{1},S_{2},S_{3}$ on the left side in Figure 3 are more likely to be misclassified, while the distance among articles $a_{1},a_{2},a_{3}$ increases (as shown on the right side in Figure 3), the boundaries of different article labels will become clearer.

(12) $$L_{p}=-\left(\frac{1}{m}\sum_{i=1}^{m}\log\frac{1}{\sum_{j=1}^{m}\;exp\;d\left(P_{a_{i}},P_{a_{j}}\right)/\alpha }+\frac{1}{n}\sum_{i=1}^{n}\log\frac{1}{\sum_{j=1}^{n}\;exp\;d\left(P_{c_{i}},P_{c_{j}}\right)/\beta }\right)$$

where α and β are adjustment coefficients that control the smoothness of the prototypical distance distribution *d*. If they are set too large, it will make the distribution smoother. Conversely, if they are set too small, it will make the distribution more concentrated and peak.

### 3.6. Training

Our final training goal is to minimize the following loss function L, which consists of three parts, i.e., the loss $L_{j}$ generated by the main task LJP, the loss $L_{t}$ generated by the head-tail set classification auxiliary task, and the prototype-prototype loss $L_{p}$.

(13) $$\begin{array}{cc} L & =L_{t}+L_{j}+L_{p} \end{array}$$

(14) $$\begin{array}{cc} L_{t} & =L(y_{t},{\hat{y}}_{t}) \end{array}$$

(15) $$\begin{array}{cc} L_{j} & =L_{a}\left(y_{a},{\hat{y}}_{a}\right)+L_{c}\left(y_{c},{\hat{y}}_{c}\right) \end{array}$$

## 4. Experiments

In this section, we conduct extensive experiments on two real-world datasets to show the effectiveness of our approach and present a detailed analysis.

### 4.1. Datasets Preparation

We use the publicly available dataset in experiments, i.e., CAIL-small and CAIL-big [23]. Following the previous data processing method [4,6,14,22], we remove cases with less than 10 meaningful words and with multiple charges or legal articles. Whereas we retain cases with a frequency less than 100 and construct a validation set for the CAIL-Big data set at a ratio of 9:1. In addition, we also remove duplicate cases in the dataset. The detailed statistics are shown in Table 2.

### 4.2. Baselines

We compare the proposed model with the following strong baselines for document classification where all single-task neural network models are trained in a multi-task framework.

- **SVM + Word2vec**: use SVM [24] to classify the text represented by the word2vec [25].
- **HAN** [10]: a hierarchical attention network for document encoding consists of word-level and sentence-level.
- **Prototypical Network** [7]: classify by computing the distance to each class prototype.
- **MultiResCNN** [11]: a multi-filter residual convolutional neural network that consists of a multi-filter convolutional layer and a residual convolutional layer.

Furthermore, we also compare the proposed model with the following representative LJP baselines.

- **TopJudge** [4]: a topological multi-task learning model that incorporates the DAG dependencies among multiple sub-tasks of LJP.
- **LADAN** [22]: an attention-based model that employs a graph neural network to learn the distinction between confusing legal articles and further achieves to distinguish confusing charges.
- **NeurJudge** [14]: use the established articles and the results of intermediate subtasks to help separate the factual description to improve LJP.
- **CTM** [6]: some contrastive-case relations are introduced to construct case triples as input of the model for LJP.

### 4.3. Implementation Details

#### 4.3.1. Evaluation Metrics

We use four metrics for performance evaluation, including Accuracy (ACC), Macro Precision (MP), Macro Recall (MR) and Macro F1-score (F1). We mainly use ACC and F1 as the evaluation metric. Taking the article prediction task as an example, the calculation process of the evaluation metrics is as follows:

(16) $$\begin{array}{cc} MP & =\frac{1}{m}\sum_{i=1}^{m}\frac{TP_{a_{i}}}{TP_{a_{i}}+FP_{a_{i}}} \end{array}$$

(17) $$\begin{array}{cc} MR & =\frac{1}{m}\sum_{i=1}^{m}\frac{TP_{a_{i}}}{TP_{a_{i}}+FN_{a_{i}}} \end{array}$$

(18) $$\begin{array}{cc} F1 & =\frac{1}{m}\sum_{i=1}^{m}\frac{2\times P_{a_{i}}\times R_{a_{i}}}{P_{a_{i}}+R_{a_{i}}} \end{array}$$

(19) $$\begin{array}{cc} ACC & =\frac{1}{N}\sum_{i=1}^{N}1\left({\hat{y}}_{i}^{a}=y_{i}^{a}\right) \end{array}$$

where $TP_{a_{i}}$, $FP_{a_{i}}$, and $FN_{a_{i}}$ are the number of true positives, false positives, and false negatives corresponding to article $a_{i}$, respectively.

#### 4.3.2. Parameter Settings

We employ Thulac (http://thulac.thunlp.org/, accessed on 8 January 2023) for Chinese segmentation. For the SVM + Word2vec model, we use the RBF kernel function. For the HAN-based models, we set the maximum number of sentences in the document to 15 and the maximum sentence length to 100. The hidden size is set to 256. For the CNN-based models, we set the maximum document length to 512, the filter size to 50 and the convolution windows size to (3,5,7,9). For the Prototypical-based model, we initialize the prototype space with Glorot initialization [26] if without article-enhanced.

During training, we employ Adam [27] as the optimizer, setting the learning rate to 1 × 10${}^{-4}$ and the weight decay to 1 × 10${}^{-4}$. We set the batch size to 128, the epoch to 20, and use an early stop mechanism with patience to 5. For the variables in LPN, we set μ to 0.4, α to 1, and β to 0.5. All baselines are implemented on Tensorflow 1.15.0 (https://www.tensorflow.org/, accessed on 8 September 2022) or Pytorch 1.10.0 (https://pytorch.org/, accessed on 8 September 2022) by referring to their source code and parameter settings. The configuration of the detailed running environment is shown in the Appendix A.1.

### 4.4. Result Analysis

#### 4.4.1. Comparison with Baselines

The experimental results on the two datasets are shown in Table 3 and Table 4 respectively, from which we have the following observations: (1) The LJP baselines (i.e., TopJudge, LADAN, NeurJudge, CTM) are overall better than the general document classification baselines (SVM + Word2vec, HAN, Prototypical, MultiResCNN) combined with the multi-task framework. On the CAIL-Small, MultiResCNN + MTL is the best among generic classification baselines, and the average F1-score of two tasks exceeds HAN + MTL 3.43% and Prototypical + MTL 4.6%, even slightly surpassing LADAN and NeurJudge. All LJP baselines significantly outperform all general classification baselines except MultiResCNN + MTL. On the CAIL-Big, however, the lead of the LJP baselines wanes, with only CTM surpassing all general classification baselines. We speculate that this is because the general model narrows the gap to the domain model moderately when given more sufficient data. (2) LPN and CTM outperform other LJP methods by a large margin. On the CAIL-Small, their average F1-scores of two tasks are 3.72% and 4.95% higher than the best model, MultiResCNN + MTL, respectively. Especially on the CAIL-Big, they lead even more, with average F1-scores of 6.76% and 7.89% higher than the best model HAN + MTL, respectively. What LPN and CTM have in common is they both set the category decoder to fuse the frequency information of labels. In addition, the data distribution of CAIL-Big is more uneven than that of CAIL-Small. It indicates that LPN and CTM are excellent in handling samples with imbalanced distribution, specifically, the category decoder of LPN and CTM works. (3) LPN performs best on average F1 metric, outperforming CTM by a small margin of 1.23% on CAIL-Small and 1.13% on CAIL-Big. However, we must point out that the input of CTM is a triplet composed of three cases, which require three times the size of training data to participate in the training, and takes longer.

#### 4.4.2. Comparison on Few-Shot Cases

**Figure 4:**
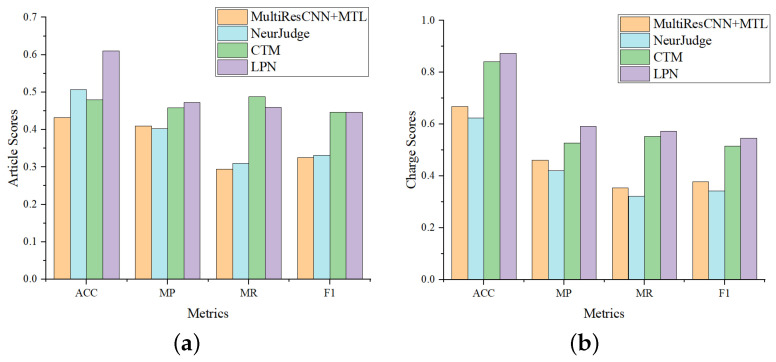
Comparison of LJP results on the tail dataset. Article scores and charge scores refer to the metrics score of the relevant article prediction and charge prediction, respectively. (**a**) Comparison of relevant article prediction. (**b**) Comparison of charge prediction.

To further evaluate the potential of LPN in predicting few-shot cases, we evaluate the model performance on the “tail set” (More than 95% of the data have label frequencies less than 100.) and compare with MultiResCNN, NeurJudge and CTM, which all perform well on CAIL-Small. Experiment results are shown in Figure 4, from which we have the following observations: (1) LPN significantly outperforms MultiResCNN and NeurJudge on all four metrics, while slightly ahead of CTM, consistent with Table 3, indicating that CTM is a strong baseline. (2) Compared with the results on CAIL-Small, it can be seen that the overall performance of all models on the “tail set” is lower than that on CAIL-Small. (3) Furthermore, we also find that LPN leads more on the “tail set”. On the CAIL-Small, the F1 of LPN on article prediction achieves 5.06% and 4.6% higher than that of MultiResCNN and NeurJudge, respectively. While on the few-shot data, LPN is 12.06% and 11.45% higher, respectively. Comparing the result of the charge prediction, LPN also leads more in the “tail set”. This verifies that LPN is better at handling few-shot legal cases than baselines.

#### 4.4.3. Curves Variation of Loss and Metrics

**Figure 5:**
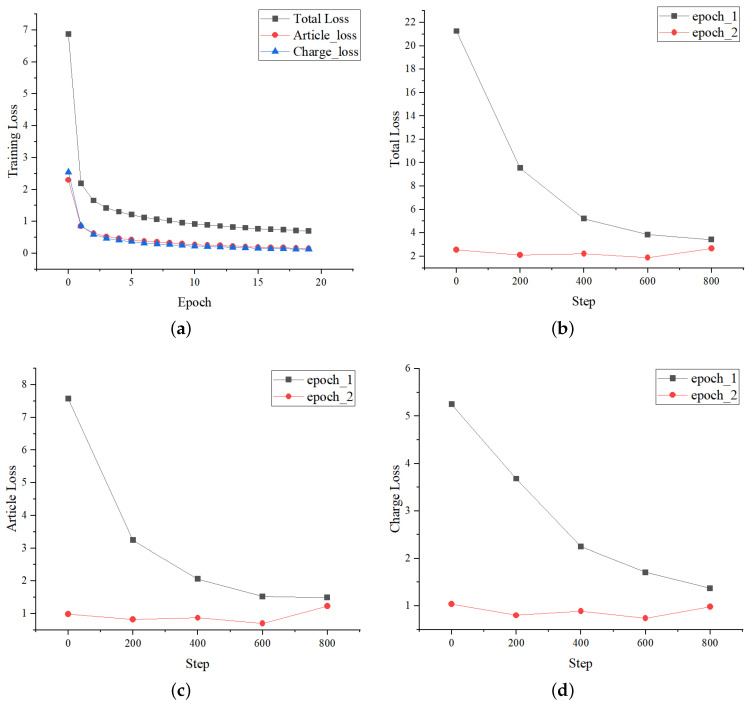
Loss variation during LPN training. The total loss is L, article loss refers to the loss $L_{a}$ of the relevant article prediction task, and correspondingly, the charge loss refers to the loss $L_{c}$ of the charge prediction task. (**a**) Loss curves with the number of epochs. (**b**) Total loss curves with the number of steps. (**c**) Article loss curves with the number of steps. (**d**) Charge loss curves with the number of steps.

The loss curves changing during the training process on CAIL-Small are shown in Figure 5. Figure 5a shows the variation curves of losses with the number of epochs. It can be seen that the losses in the first two epochs have a significant decline, and the losses tend to be stable within 5 epochs. Furthermore, the variation of $L,L_{a},L_{c}$ is consistent. The specific changes of every 200 batches in the first two epochs are shown in Figure 5b–d, where the curves of different losses with the number of steps changes are consistent too. The above indicates that the loss function setting of LPN is effective.

**Figure 6:**
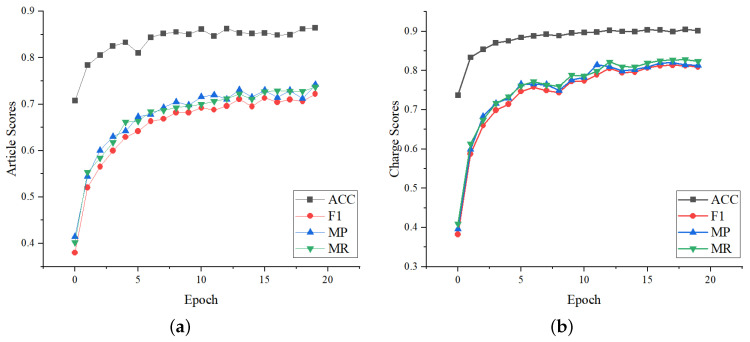
Metrics score variation on the validation set during LPN training. Article scores and charge scores refer to the metrics score of the relevant article prediction and charge prediction, respectively. (**a**) Article metrics curves with the number of epochs. (**b**) Charge metrics curves with the number of epochs.

We further evaluate the robustness performance of LPN, whose metrics variation on the validation set during training are shown in Figure 6. It can be seen that the changes in the article metrics and the charge metrics are consistent. They all rise rapidly in the first five epochs, then rise slowly with fewer fluctuations, and stablize after 15 epochs. It shows that the setting of LPN is reasonable.

#### 4.4.4. Ablation Study on CAIL-Small

To further analyze the role of each module in LPN, we conduct an ablation study on CAIL-Small, the results are shown in Table 5. Specifically, we first consider removing the category decoder (denoted as -CD), i.e., keeping only one prototype decoder without distinguishing head/tail sets. Next, we remove the article description module (denoted as -AE), i.e., randomly generating each article prototype. Then we remove the prototype-prototype loss (denoted as -PPL), i.e., the loss function of LPN in Equation (13) degenerates to $L=L_{t}+L_{j}$. Finally, we remove the head prototype module (denoted as -HP) and tail prototype module (denoted as -TP) respectively, i.e., the prediction results are directly calculated after passing through the corresponding multi-task decoder (as shown in Equations (5)–(7)).

Experimental results show that the performance of LPN decreases when any module is removed, indicating the effectiveness of these modules. -CD drops substantially, indicating that the category decoder module plays an important role in LPN. After all, the more balanced the category distribution of data, the more conductive to the learning of prototype representation. -HP also has a slight decrease, indicating that LPN improves the prediction ability not only for few-shot cases but also for overall cases. Comparing -TP with -HP, -TP drops more, indicating that the impact of the tail prototype module is greater than that of the head prototype module. It is consistent with our conjecture that the prototype module can effectively improve the prediction of few-shot cases.

#### 4.4.5. Hyperparameter Optimization

**Figure 7:**
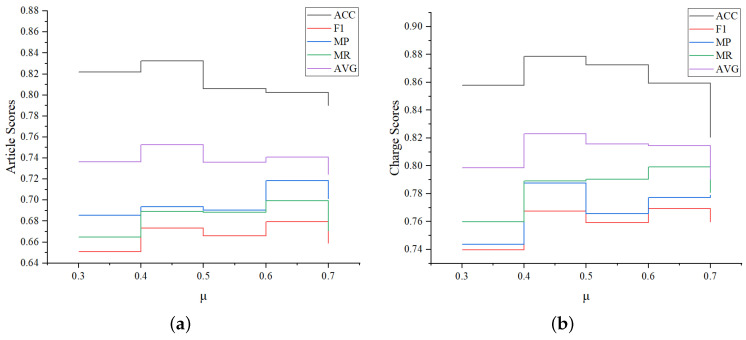
Comparison of LJP results with different hyperparameter settings. (**a**) Relevant article prediction results. (**b**) Charge prediction results.

To further evaluate the robustness of LPN, we compare its performance change under different hyperparameter settings. Since the above analysis shows that the category decoder module of LPN has a great influence, we conduct a set of experiments to compare the changes of various indicators when μ is 0.3, 0.4, 0.5, 0.6, 0.7 respectively. The experimental results are shown in Figure 7. We found that the overall changes of MP, MR, and F1 are consistent, achieving the maximum value at μ = 0.6. However, the ACC and AVG((ACC + F1)/2) value is the best and the overall performance of the model is the best when μ equals 0.4, then gradually decreases with the increase of μ. That’s because as μ increases, the data distribution of the “head set” becomes more convex, while the “tail set” is relatively smooth. Correspondingly, since the ACC value is greatly affected by the “head set” result, it will become worse. On the contrary, the F1 value is greatly affected by the “tail set”, and will become better.

### 4.5. Visualization of Cases

**Figure 8:**
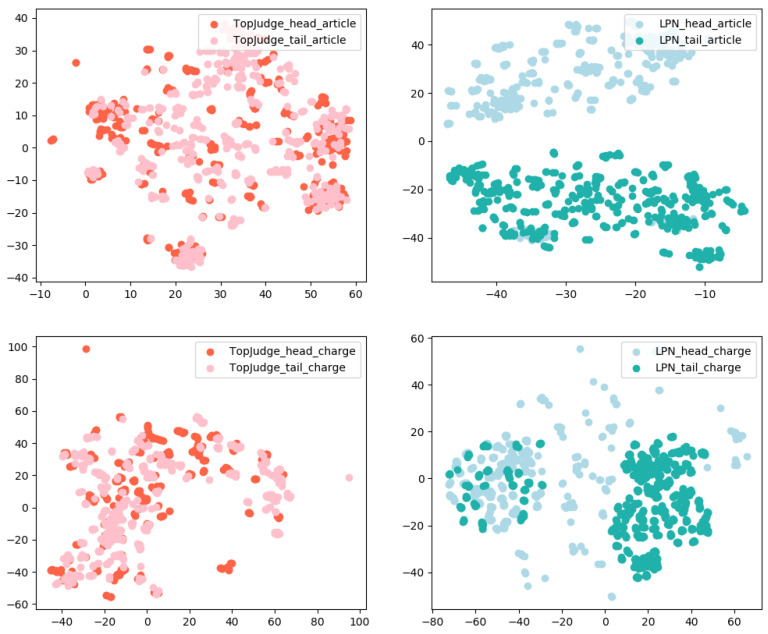
Visualization of some randomly sampled cases with high and low frequency.

We analyze the gain of category decoder from the perspective of case representations, i.e., compare the case representations generated by LPN with those generated by TopJudge. Specifically, we randomly sample 5 cases from ”head set” (or “tail set”) for each legal article and charge, forming the corresponding smaller head (or tail) case set. We respectively use t-SNE [28] to visualize the case representations generated by TopJudge and LPN on different sets. Visualization results are shown in Figure 8. It can be clearly seen that LPN can effectively distinguish the “head set” and “tail set”, indicating the effectiveness of the category decoder.

**Figure 9:**
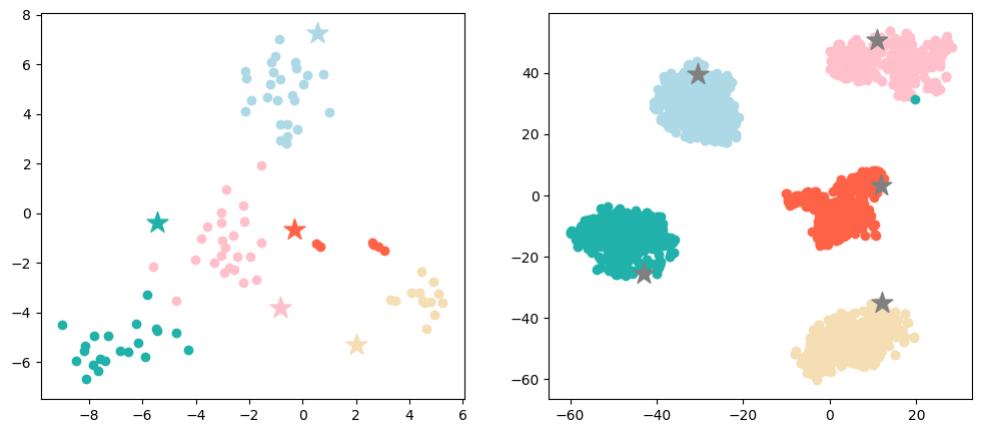
Visualization of some randomly sampled cases and their corresponding prototype representations, where circles represent cases, and five-pointed stars represent prototypes. The left figure is a sampling from the “tail set”, and the right figure is a sampling from the “head set”.

Next, we perform a visual analysis of the representation of prototypes and cases. Specifically, we randomly sample 5 prototypes and their corresponding cases from the “tail set” and “head set” constructed in Section 3.4. The representation generated by LPN is then visualized with t-SNE as shown in Figure 9, it can be seen that prototypes are well surrounded by their corresponding cases. Correspondingly, it also explains our prediction results to a certain extent, i.e., the prototype category closest to the sample is predicted. Comparing the left and right figures, it is obvious that the “head set” prototype representation is better, while there are some fuzzy points on the category boundaries on the “tail set”, and the prototype representation is not the center of the case. This is reasonable because the prototype in LPN is different from [7]. It is not simply the average value of all sample representations, is the parameter learned in the network.

## 5. Conclusions

In this paper, we propose a label-enhanced prototypical network, LPN, to alleviate the problem of model bias towards cases with high-frequency resulting from category imbalance in LJP. LPN consists of the factual description encoder, article encoder, category decoder, head prototype decoder, and tail prototype decoder. We conduct experiments on two public datasets, and the results show that our method can effectively improve the performance of LJP. That is, LPN achieves 67.32%/73.96% and 76.75%/82.67% in F1 of article prediction and charge prediction on CAIL-Small/CAIL-Big respectively, and the average F1 of the two subtasks exceeds all baselines. Similarly, LPN also outperforms other baselines on few-shot cases, verifying its excellent learning ability. However, the interpretability of LPN has certain limitation, which just gives the predicted relevant article and charge. It can explain the prediction results by staying at the model level, without giving a comprehensible and convincing explanation for ordinary people, such as “the defendant’s behavior led to the corresponding punishment”. In the future, we will explore improving the interpretability of LJP, e.g., by fusing auxiliary knowledge such as guiding cases and intermediate tasks into the model, or building a domain knowledge graph based on the pre-trained large model to enhance the explainable of LJP.

## Figures and Tables

**Table 1:** Main mathematical notations.

Notation	Description
$S_{i}^{d}=\left\{w_{1}^{d},\ldots ,w_{ld}^{d}\right\}$	the factual description of case *i*
$Y^{a}=\left\{a_{1},\ldots ,a_{m}\right\}$	the set of article labels
$Y^{c}=\left\{c_{1},\ldots ,c_{n}\right\}$	the set of charge labels
$S^{ai}=\left\{w_{1}^{ai},\ldots ,w_{la}^{ai}\right\}$	the description of article ai
$Y^{t}=\left\{0,1\right\}$	the set of category labels

**Table 2:** Statistics of the datasets.

	CAIL-Small	CAIL-Big
Training Set Cases	103,170	1,390,706
Test Set Cases	27,656	185,603
Validation Set Cases	14,232	158,761
Legal Articles	177	181
Charges	191	193

**Table 3:** Experimental results on CAIL-Small of our model and baselines. (Values in the table are percentages, and the best results are shown in bold).

Tasks	Relevant Articles				Charges				LJP
Metrics	ACC.	MP	MR	F1	ACC.	MP	MR	F1	Avg. F1
SVM + Word2vec	72.62	53.28	46.47	46.45	74.99	59.20	51.54	51.90	49.18
HAN + MTL	76.34	63.40	60.58	59.61	80.68	70.47	67.99	67.70	63.66
Prototypical + MTL	78.25	65.22	60.03	59.59	80.98	71.01	66.29	65.39	62.49
MultiResCNN + MTL	79.21	68.02	61.66	62.26	83.80	77.19	70.64	71.91	67.09
TopJudge	77.74	63.86	61.87	60.77	80.57	71.06	68.89	67.94	64.36
LADAN	77.37	65.28	63.89	62.44	82.54	73.41	71.73	71.16	66.80
NeurJudge	79.84	67.90	63.35	62.72	81.01	71.83	68.00	67.86	65.29
CTM	80.22	67.65	69.09	65.84	87.14	76.05	78.63	75.77	70.81
LPN	**83.25**	69.37	68.91	**67.32**	**87.85**	78.76	78.92	**76.75**	**72.04**

**Table 4:** Experimental results on CAIL-Big of our model and baselines. (Values in the table are percentages, and the best results are shown in bold).

Tasks	Relevant Articles				Charges				LJP
**Metrics**	**ACC.**	**MP**	**MR**	**F1**	**ACC.**	**MP**	**MR**	**F1**	**Avg. F1**
SVM + Word2vec	94.06	65.15	49.04	52.08	93.65	71.36	53.83	57.43	54.76
HAN + MTL	96.18	76.40	63.44	66.82	96.14	82.90	70.98	74.04	70.43
Prototypical + MTL	95.75	74.78	62.01	65.30	95.75	84.33	68.94	73.16	69.23
MultiResCNN + MTL	95.71	77.40	60.15	64.75	95.63	84.83	67.11	72.29	68.52
TopJudge	94.85	66.67	56.15	57.75	94.50	69.90	58.90	60.17	58.96
LADAN	95.88	73.87	64.37	67.30	95.65	80.34	69.39	72.55	69.93
NeurJudge	95.85	75.35	64.99	67.48	95.42	81.82	70.57	72.97	70.23
CTM	**96.73**	77.59	73.64	**74.54**	96.57	83.04	79.15	79.83	77.19
LPN	96.72	78.99	73.03	73.96	**96.73**	85.52	82.11	**82.67**	**78.32**

**Table 5:** Experimental results of the ablation studies on CAIL-Small. -CD refers to removing the category decoder, -AE refers to removing the article encoder, -PPL refers to removing the prototype-prototype loss, -HP refers to removing the head prototype module, -TP refers to removing the tail prototype module. (Values in the table are percentages, and the best results are shown in bold).

Tasks	Relevant Articles				Charges			
**Metrics**	**ACC.**	**MP**	**MR**	**F1**	**ACC.**	**MP**	**MR**	**F1**
-CD	78.86	67.79	62.24	62.57	84.05	72.90	69.86	69.69
-AE	81.67	67.34	68.80	66.23	87.85	76.85	77.91	75.81
-PPL	82.80	67.33	68.06	65.67	**87.86**	77.74	77.56	75.23
-HP	82.90	69.06	68.19	66.42	87.51	76.41	78.50	75.78
-TP	82.12	67.40	68.92	66.44	87.24	75.43	76.61	74.44
LPN	**83.25**	69.37	68.91	**67.32**	87.85	78.76	78.92	**76.75**

## Data Availability

The datasets used to support the findings of this study are available from https://github.com/thunlp/CAIL, accessed on 8 January 2023.

## Appendix A

### Appendix A.1. Running Environment

We run our models and baselines on the Ubuntu 22.04.2 LTS system, which has four GPUs with the vision of Tesla V100 and each has 32 Gb memory. The model name of the CPU is Intel(R) Xeon(R) Silver 4216 CPU @ 2.10 GHz, which has 32 Cores and 128 GB memory.

### Appendix A.2. Legal Article Description

The description corresponds to legal article NO.244, which contains a lot of valuable information, such as the definition of illegal acts, the basis for sentencing, etc.

**Table A1:** Description of legal article 244.

Legal Article	Description
NO.244	[Crime of forced labor] (1) Whoever forces others to work by means of violence, threats, or restriction of personal freedom shall be sentenced to fixed-term imprisonment of not more th- an three years or criminal detention and shall also be fined; if the circumstances are serious, he shall be sentenced to fixed-term imprisonment of not less than th- ree years but not more than ten years and shall also be fined. (2) Whoever knows that others have committed the acts in (1), but still recruiting or transporting personnel for them, or assisting and forcing others to work, shall be punished following the provisions in (1). (3) Units that commit the crimes mentioned in (1) and (2) shall be fined, where th- e directly responsible managers and other directly responsible personnel shall be punished following the provisions in (1).
